# Defining Composition–Cytokine Relationships Enables the Design of Lipid Nanoparticles with Programmed Immunogenicity

**DOI:** 10.1002/smll.74283

**Published:** 2026-07-20

**Authors:** Adam Alexander Walters, Belal I. Hanafy, Chuan‐En Lu, Sara Marelli, Alaa Zam, Kai Liu, Rachel E. Foreman, Ramesh Soundararajan, Philippe Collin, Lennart Lindfors, Alan Sabirsh, Mariarosa Mazza, Khuloud T. Al‐Jamal, Stephanie M. Bates, George Thom, Jan Rehwinkel, Alexander N Kapustin

**Affiliations:** ^1^ Advanced Drug Delivery Pharmaceutical Sciences BioPharmaceuticals R&D AstraZeneca Cambridge UK; ^2^ Advanced Drug Delivery Pharmaceutical Sciences BioPharmaceuticals R&D AstraZeneca Gothenburg Sweden; ^3^ Discovery Sciences BioPharmaceuticals R&D AstraZeneca Cambridge UK; ^4^ Institute of Pharmaceutical Science School of Cancer & Pharmaceutical Sciences King's College London London UK; ^5^ Discovery Bioanalysis Clinical Pharmacology and Safety Sciences BioPharmaceuticals R&D AstraZeneca Cambridge UK; ^6^ Cell Therapy Safety Clinical Pharmacology and Safety Sciences BioPharmaceuticals R&D AstraZeneca Cambridge UK; ^7^ Department of Pharmacology and Pharmacy, Li Ka Shing Faculty of Medicine The University of Hong Kong Hong Kong China; ^8^ Immune Safety Clinical Pharmacology and Safety Sciences BioPharmaceuticals R&D AstraZeneca Cambridge UK; ^9^ MRC Translational Immune Discovery Unit MRC Weatherall Institute of Molecular Medicine Radcliffe Department of Medicine University of Oxford Oxford UK

**Keywords:** composition–cytokine relationship, innate immunity, ionizable lipids, lipid nanoparticles, mRNA vaccines, PEGylated lipids

## Abstract

Lipid nanoparticles (LNPs) are central to next‐generation vaccines, yet candidate selection remains largely empirical, limiting early identification of formulations associated with rare adverse events such as myocarditis. A better understanding of LNP composition–immunogenicity relationships is therefore critical for rational vaccine design. Here, we profiled a panel of clinically relevant LNP formulations across complementary in vitro and in vivo models to define mechanisms underlying innate immune activation and adaptive responses. We identified three distinct cytokine programs: (i) a monocyte chemoattractant protein‐1 (MCP‐1)‐dominated inflammatory response associated with cytotoxic stress; (ii) inflammasome‐dependent interleukin‐1 beta (IL‐1β) secretion requiring pro‐inflammatory priming; and (iii) type I and II interferon‐dependent responses in which LNPs synergize with interferon gamma (IFNγ) to amplify interferon gamma‐induced protein 10 (IP‐10) production. Using a design of experiments (DoE) framework with formulation feature analysis, we found that polyethylene glycol‐conjugated (PEGylated) lipid content and ionizable lipid identity are key modulators of the IFNγ/IP‐10 axis, previously implicated in vaccine‐associated myocarditis. In vivo validation showed that innate cytokine responses are strongly influenced by lipid composition, whereas adaptive humoral and cellular responses correlate with transgene expression rather than cytokine magnitude. Collectively, these findings define relationships among LNP composition, cytokine induction, and vaccine efficacy, and provide a framework for rational design and screening of LNP‐based vaccines that maximize immunogenicity while minimizing reactogenicity.

## Graphical abstract

Lipid nanoparticle composition governs distinct cytokine programs through the interplay of ionizable and polyethylene glycol‐conjugated (PEGylated) lipids. In vitro profiling, design of experiments (DoE) modeling, and in vivo validation reveal how formulation can tune reactogenicity‐associated pathways, while transgene expression remains the strongest predictor of adaptive immunity.

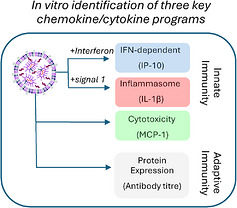

## 1 Introduction

Recent advances in lipid nanoparticle (LNP)‐mediated mRNA delivery have transformed vaccine development and enabled a broad range of therapeutic applications, from gene editing to in vivo chimeric antigen receptor T‐cell (CAR‐T) generation and therapeutic protein expression [1]. The success of LNP technology derives from its carefully optimized composition: ionizable lipids, helper lipids such as 1,2‐distearoyl‐sn‐glycero‐3‐phosphocholine (DSPC), cholesterol, and polyethylene glycol‐conjugated (PEGylated) lipids, which together ensure efficient and well‐tolerated intracellular mRNA delivery [2]. Among these, ionizable lipids have been pivotal to the clinical adoption of LNPs. Neutral at physiological pH, they improve biocompatibility, yet acquire a positive charge in acidic endosomal compartments, facilitating nucleic acid complexation and endosomal escape. Beyond delivery, ionizable lipids have been shown to drive the adjuvant activity of LNPs, a property critical for vaccine efficacy [3, 4, 5, 6, 7]. This adjuvant effect is supported by studies in which co‐administration of LNPs with soluble antigen elicited immune responses, even when the mRNA itself was chemically modified to be immunologically inert or absent entirely [5, 6, 8, 9, 10]. More recently, PEGylated lipids have been implicated in modulating LNP immunogenicity, indicating that the adjuvant activity of LNPs is driven by both ionizable and PEGylated components [11, 12]. Severe acute respiratory syndrome coronavirus 2 (SARS‐CoV‐2) vaccination using LNPs demonstrated outstanding efficacy with acceptable tolerability, yet adverse events ranging from mild local inflammation to rare cytokine‐driven myocarditis underscore the need for improved predictive understanding of LNP‐driven immunogenicity [13, 14].

The rapid adoption of mRNA‐LNP platforms during the COVID‐19 pandemic has intensified focus on the mechanisms underlying LNP‐driven immunogenicity and vaccine efficacy. Vaccinology studies consistently report activation of innate immune cells—primarily myeloid‐lineage cells—and upregulation of cytokines such as interleukin‐15 (IL‐15), interferon gamma (IFNγ), and interferon gamma‐induced protein 10 (IP‐10; also known as C‐X‐C motif chemokine ligand 10) following immunization [15]. Elevated IFNγ/IP‐10 signatures correlate positively with vaccine‐induced antibody responses and overall efficacy, whereas reduced expression of these cytokines in elderly humans and mice is associated with impaired humoral immunity [16, 17, 18]. Preclinical studies in rats have shown that LNPs formulated with the ionizible lipids SM‐102 or MC3 robustly induce IP‐10, further underscoring the central role of the IFNγ/IP‐10 axis in LNP immunogenicity across species, yet the mechanisms by which LNPs activate these pathways remain poorly defined [19, 20]. Concurrently, SM‐102‐ and MC3‐based LNPs have been reported to activate the inflammasome, triggering interleukin‐1 beta (IL‐1β) and interleukin‐18 (IL‐18) release, pleiotropic mediators implicated in reactogenicity [7, 21]. The heterogeneity of cytokine responses observed across LNP studies likely reflects variability in innate sensing mechanisms among immune cells: LNPs can activate the CD1d receptor on natural killer T (NKT) cells, stimulate nuclear factor kappa‐light‐chain‐enhancer of activated B cells (NF‐κB) downstream of Toll‐like receptor 4 (TLR4) in monocytes, and engage additional innate lymphoid and myeloid populations [3, 4, 5]. These effects are distinct from canonical adaptive responses, yet other pro‐inflammatory cytokines, such as interleukin‐6 (IL‐6), can be induced independently of myeloid differentiation primary response 88 (MyD88)‐ or mitochondrial antiviral‐signaling protein (MAVS)‐dependent pathways, while remaining important for humoral immunity. For example, melanoma differentiation‐associated protein 5 (MDA5) knockout profoundly impacts T cell responses [22], and the interplay between T cells and monocytes has recently been linked to IFNγ/IP‐10‐driven myocarditis [23]. Notably, Cao et al. recently demonstrated that pharmacologic inhibition of the IFNγ/IP‑10 axis ameliorates myocarditis in a preclinical SARS‐CoV‐2 mRNA vaccine model [23]. Together, these studies highlight the complexity of cytokine responses to LNPs and the need to understand composition–cytokine relationships across model systems.

Here, we profiled a panel of commercially relevant LNPs with favorable safety profiles to identify composition–cytokine relationships, focusing on clinically relevant pathways including general inflammatory cytokines, inflammasome activation, and interferon‐dependent signaling. Using a design of experiments (DoE) approach integrated with machine‐learning analysis, we identified the combination of PEGylated and ionizable lipids as key modulators of cytokine programs and revealed strong synergy between environmental context and pathway‐specific responses. Following in vitro profiling, LNPs were tested in vivo using the identified metrics as readouts. Correlation analysis demonstrated that the magnitude of innate cytokine responses is not predictive of adaptive efficacy, whereas antibody titers correlate most closely with in vivo transgene expression.

## 2 Results and Discussion

### 2.1 Immunogenicity is Linked to the Type of Ionizable Lipid and is Independent of Protein Expression

Lipid nanoparticles (LNPs) provide an inherent adjuvant effect that enhances vaccine immunogenicity, however, excessive cytokine production and reactogenicity have been linked to rare adverse events, including fever and myocarditis [5, 6, 7, 14, 15]. Although recent studies have shown that ionizable lipid–driven endosomal escape can activate inflammatory signaling pathways, the mechanisms by which LNPs engage innate immune sensors and shape downstream adaptive responses remain incompletely defined. This knowledge gap likely reflects both the composite architecture of LNP formulations and the complexity of immune sensing across diverse tissues and cell types [24, 25, 26, 27].

**FIGURE 1:**
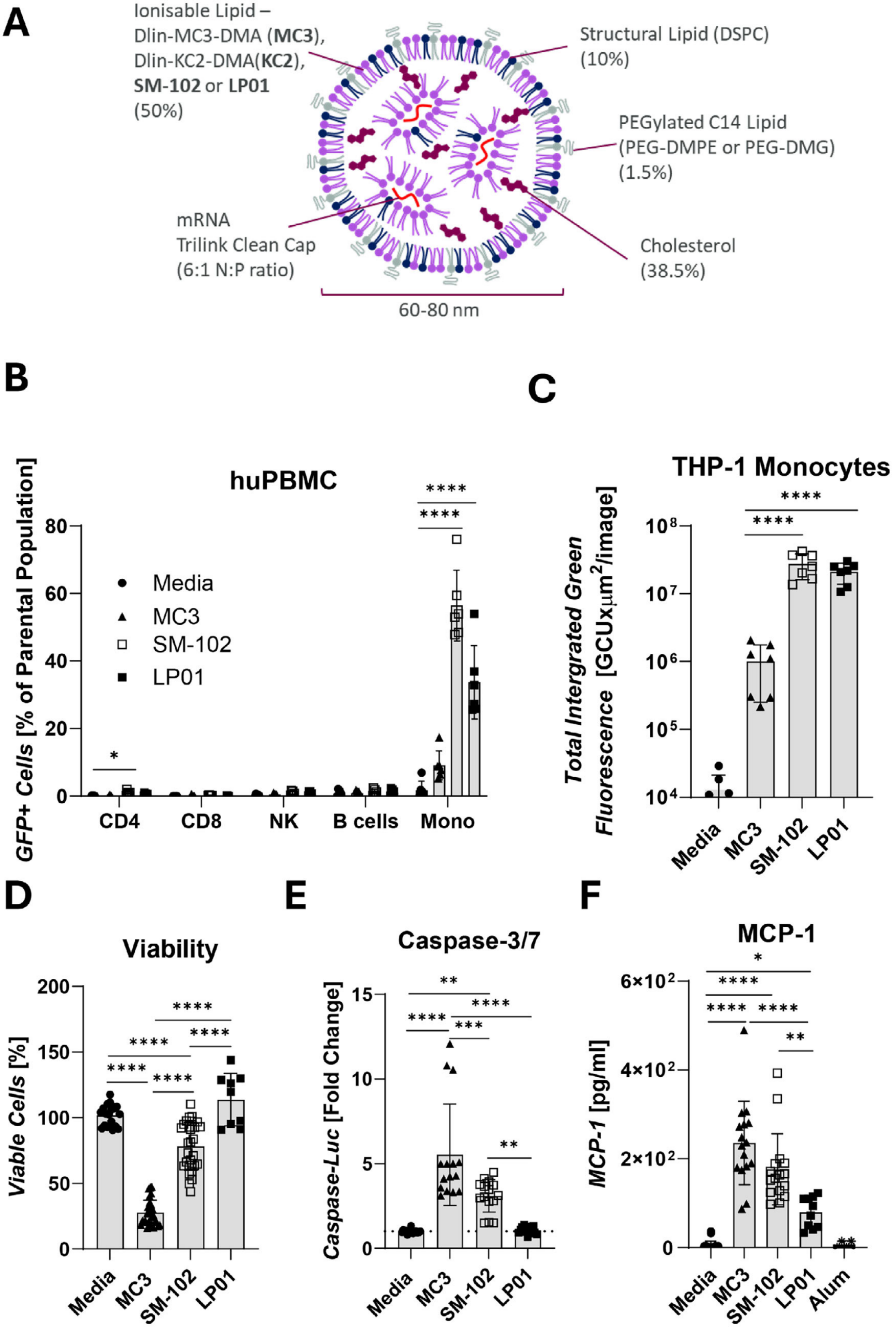
LNPs deliver mRNA to primary monocytes and a monocytic cell line, resulting in ionizable lipid‐dependent cytotoxicity and cytokine release. (A) Schematic of the standard LNP formulation used in the study (B) Human PBMCs were incubated with GFP LNPs (MC3, SM‐102, or LP01) for 48 h, stained for viability and major immune cell markers, including CD4+ T cells (CD4+, CD3+), CD8+ T cells (CD8+, CD3+), NK cells (CD56+, CD3−), B cells (CD19+, CD3−), and monocytes (CD14+), and analyzed by flow cytometry; GFP+ cells are presented as a percentage of each cell subset. Bars represent mean ± SD; *n* = 5 donors; individual data points represent individual donors, with one donor analyzed in technical duplicate. Data were analyzed using Student's *t*‐test. (C) THP‐1 cells (50 000/well) were treated with 1 µg/mL LNPs formulated with MC3, SM‐102, or LP01. After 48 h, GFP expression was quantified using Incucyte image analysis and plotted as total integrated green fluorescence per well. Bars represent mean ± SD; *n* = 3 independent experiments; individual data points represent technical replicates pooled across experiments. Data were analyzed using one‐way ANOVA. (D) Cell viability after 48 h of LNP exposure was assessed using CellTiter‐Glo and is shown as the percentage of viable cells relative to media‐treated controls. Bars represent mean ± SD; *n* ≥ 3 independent experiments; individual data points represent technical replicates pooled across experiments. Data were analyzed using one‐way ANOVA. (E) Caspase‐3/7 activity was measured after 48 h using the Caspase‐Glo assay and is presented as fold change relative to media. Bars represent mean ± SD; *n* = 5 independent experiments; individual data points represent technical replicates pooled across experiments. Data were analyzed using one‐way ANOVA. (F) Supernatants from THP‐1 stimulations were collected and analyzed for MCP‐1 by ELISA. Bars represent mean ± SD; *n* = 7 independent experiments for MC3 and SM‐102, and *n* = 4 independent experiments for LP01; individual data points represent technical replicates pooled across experiments. Data were analyzed using one‐way ANOVA. ^*^ *p* < 0.05, ^**^ *p* < 0.01, ^***^ *p* < 0.001, ^****^ *p* < 0.0001.

Here, we profiled a commercially relevant panel of ionizable lipids developed for vaccine, RNA interference, and gene‐editing applications [28, 29, 30]. LNPs were formulated by microfluidic mixing at a 50:38.5:10:1.5 molar ratio of ionizable lipid, cholesterol, structural lipid, and PEGylated lipid (Figure 1A), yielding particles with comparable size, polydispersity index (PDI), and encapsulation efficiency (Table S1). The selected lipids span a range of reported biodegradability, from higher for LP01 and SM‐102 to lower for MC3 [28, 29, 31, 32], allowing us to compare ionizable lipids with distinct physicochemical and biological properties.

Whole human peripheral blood mononuclear cells (PBMCs) have been used previously to profile LNP immunogenicity [7], however, PBMC‐based models suffer from substantial donor‐to‐donor variability, limited scalability, and logistical constraints related to donor recruitment, ethical approval, and complex isolation procedures. To identify a suitable in vitro surrogate model, we first assessed LNP transfection across immune subsets within PBMCs using flow cytometry. A phenotyping panel was used to identify CD4^+^ T cells (CD3^+^CD4^+^), CD8^+^ T cells (CD3^+^CD8^+^), natural killer (NK) cells (CD3^−^CD56^+^), B cells (CD3^−^CD19^+^), and monocytes (CD14^+^) (Figure 1B). Robust green fluorescent protein (GFP) expression was detected exclusively in CD14^+^ monocytes, with MC3 achieving lower transfection efficiency (14%) compared with the more biodegradable third‐generation lipids SM‐102 and LP01 (56% and 33%, respectively).

Given that monocytes were the only PBMC subset that showed clear LNP association and payload expression, we next assessed whether a clinically relevant cytokine profile could be recapitulated using an in vitro THP‐1 monocytic model [5]. GFP mRNA‐formulated LNPs were efficiently expressed in THP‐1 cells, with SM‐102 and LP01 yielding approximately tenfold higher expression than MC3, and no significant difference between SM‐102 and LP01, consistent with PBMC observations (Figure 1C).

As cell death–associated release of inflammatory mediators is a known driver of innate immune activation, we hypothesized that general inflammatory responses would correlate with cytotoxic stress. MC3‐containing LNPs reduced THP‐1 viability by approximately 70%, compared with a 20% reduction for SM‐102 (Figure 1D). Consistent with this, MC3 induced a pronounced fivefold increase in caspase‐3/7 activity, while SM‐102 induced an intermediate threefold response, LP01 showed no detectable cytotoxicity or caspase activation (Figure 1E). Initial multiplex cytokine screening (data not shown) identified monocyte chemoattractant protein‐1 (MCP‐1) as the cytokine most strongly and consistently associated with cytotoxicity, following the trend MC3> SM‐102> LP01. This was confirmed in follow‐up experiments, where MC3 and SM‐102 induced MCP‐1 levels of 235 and 176 pg/mL, respectively, significantly higher than LP01 (79 pg/mL) (Figure 1F). IP‐10 was modestly but significantly increased in MC3 and SM‐102 groups (53 and 33 pg/mL, respectively; data not shown), while IL‐1β remained undetectable across all conditions.

Fast biodegradability of SM‐102 and LP01 and slow MC3 hydrolysis were established across several in vivo studies as well as a cell‐free assay [28, 29, 31, 32]. To assess intracellular lipid biodegradability in vitro, we isolated cytosolic and mitochondria‐enriched fractions from THP 1 cells 24 h after LNP treatment and detected ionizable lipids in both (Figure S1A,B). Notably, MC3 accumulated at least three‐fold more than LP01 and SM‐102. This distribution is consistent with prior in vivo reports of faster biodegradation and/or clearance for LP01 and SM‐102 relative to MC3 [28, 29, 31]. It is plausible that reduced biodegradability contributes to MC3‐associated cytotoxicity. Altogether, these results indicate that inflammatory cytokine induction, particularly MCP‐1, correlates with cytotoxic stress rather than protein expression. In our experiments, MC3 elicited pronounced caspase‐3/7 activation and MCP‐1 release. Although our data strongly support a link between ionizable lipid biodegradability, cytotoxicity, and inflammatory signaling, further work should define the contributions of headgroup/side chain and tail chemistry, metabolic by‐products, and subcellular partitioning, and assess additional determinants of cytotoxicity and immunogenicity.

### 2.2 In Vitro LNP Profiling Reveals Context‐Dependent Immunogenicity

To define composition–cytokine relationships, we focused on cytokine programs implicated in clinical and pre‐clinical studies, including general inflammation (IL‐6 and MCP‐1), inflammasome activation (IL‐1β), and interferon‐dependent responses (IP‐10) [6, 7, 15]. However, in this model, we did not observe substantial IL‐1β or IP‐10 responses under basal conditions, despite these cytokines being key readouts in both pre‐clinical and clinical settings [7, 17, 19]. This indicates that the in vitro THP‐1 model may not fully recapitulate the inflammatory milieu present in vivo. Mechanistically, IL‐1β production requires activation of the classical two‐signal inflammasome pathway, whereas IP‐10 is an interferon‐stimulated gene secreted downstream of interferon signaling [33, 34].

**FIGURE 2:**
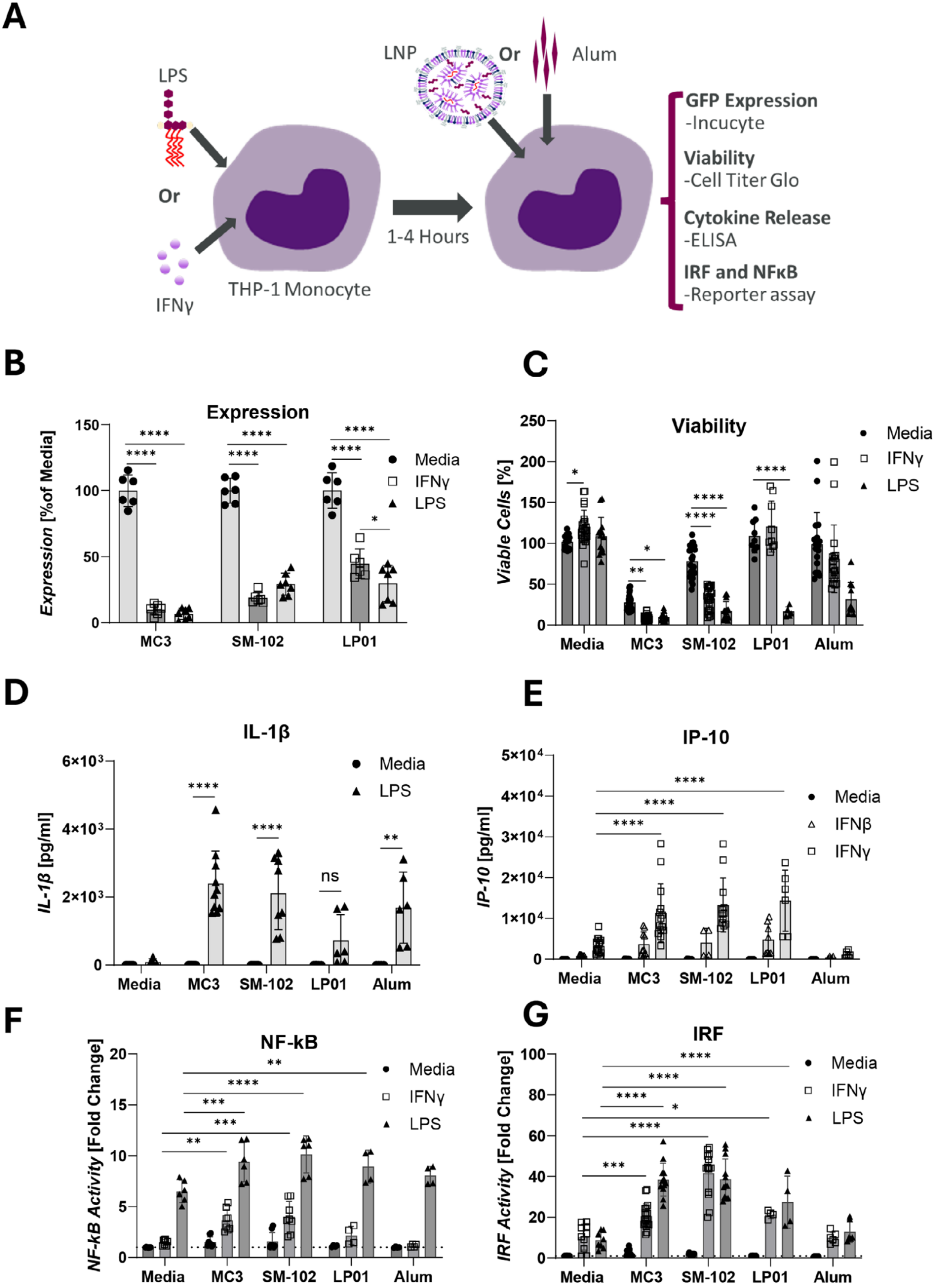
LNPs synergize with LPS and IFN to induce IL‐1β and IP‐10, respectively. (A) To model context‐specific cytokine release, THP‐1 cells (50 000/well) were exposed to media, LPS (1 µg/mL, 4 h), or IFN (IFNβ or IFNγ, 20 ng/mL, 1 h) before addition of eGFP LNPs (1 µg/mL) formulated with MC3, SM‐102, or LP01. (B) Following a further 48 h incubation, GFP expression was quantified using Incucyte and plotted as total integrated green fluorescence as a percentage of the media‐only condition. Bars represent mean ± SD; *n* = 3 independent experiments; individual data points represent technical replicates pooled across experiments. Data were analyzed using two‐way ANOVA. (C) Cell viability was assessed using CellTiter‐Glo and is shown as the percentage of viable cells relative to the media‐treated control. Bars represent mean ± SD; *n* ≥ 3 independent experiments; individual data points represent technical replicates pooled across experiments. Data were analyzed using two‐way ANOVA. Aluminum hydroxide (Alum, 100 µg/mL) served as a non‐lipid‐based vaccine adjuvant. (D, E) To determine cytokine levels, supernatants were assessed using MSD U‐Plex or DuoSet ELISA kits, with measurements acquired using MSD or EnVision plate readers. Data for IL‐1β are shown in (D), and data for IP‐10 are shown in (E). For IL‐1β, *n* = 5 independent experiments for MC3, *n* = 4 independent experiments for SM‐102, and *n* = 3 independent experiments for LP01. For IP‐10, *n* = 3–6 independent experiments for IFNγ conditions and *n* = 2–5 independent experiments for IFNβ conditions. Bars represent mean ± SD; individual data points represent technical replicates pooled across experiments. Data were analyzed using two‐way ANOVA. (F, G) Supernatants were also assayed for NF‐κB and IRF reporter activity. NF‐κB activity was determined by mixing 20 µL supernatant with 180 µL Quanti‐Blue, incubating for 45 min at 37°C, and measuring OD620 nm (F). IRF activity was measured by adding 10 µL supernatant to 50 µL Quanti‐Luc and recording luminescence (G). Fold change was calculated relative to media controls (dotted line). For NF‐κB, *n* = 3–4 independent experiments for MC3 and SM‐102, and *n* = 2 independent experiments for LP01. For IRF, *n* = 6–9 independent experiments for MC3 and SM‐102, and *n* = 2 independent experiments for LP01 and Alum. Bars represent mean ± SD; individual data points represent technical replicates pooled across experiments. Data were analyzed using two‐way ANOVA. ^*^ *p* < 0.05, ^**^ *p* < 0.01, ^***^ *p* < 0.001, ^****^ *p* < 0.0001.

To better interrogate these pathways, we assessed the immunogenicity of LNPs containing distinct ionizable lipids in THP‐1 cells pre‐stimulated with either lipopolysaccharide (LPS) or interferon to model an inflammatory context (Figure 2A). In all cases, LPS pre‐stimulation reduced both protein expression and cellular viability (Figure 2B,C), consistent with previous reports [33]. The reduction in viability may reflect pyroptotic cell death, while suppressed protein expression likely results from translational arrest associated with type I interferon induction following LPS exposure. Pre‐stimulation with IFNγ similarly reduced GFP expression across all LNP formulations (Figure 2B). Notably, synergistic cytotoxicity was observed for SM‐102 and MC3, but not LP01, following IFNγ exposure (Figure 2C).

In an inflammasome‐sensitized model, THP‐1 cells were primed with LPS for 4 h prior to LNP addition. Under these conditions, MC3 and SM‐102 induced comparable levels of IL‐1β secretion (Figure 2D). Aluminum hydroxide (Alum) also triggered IL‐1β release, consistent with its role as a canonical inflammasome signal 2. Similar trends were observed in murine splenocytes and human PBMCs (data not shown). LP01, however, induced substantially lower IL‐1β secretion than the less biodegradable ionizable lipids (mean 720 pg/mL vs. >2000 pg/mL). In contrast to LPS, IFNγ pre‐conditioning did not induce IL‐1β secretion, whereas tumor necrosis factor alpha (TNF‐α) pre‐treatment, included as a positive control, produced a measurable response (Figure S2A).

These findings indicate that inflammasome‐dependent IL‐1β secretion requires synergy between ionizable lipid–mediated cellular stress and pre‐existing pro‐inflammatory signals. Although SM‐102 LNP‐mRNA has been reported to induce IL‐1β secretion in PBMCs, where LNPs were proposed to provide both priming (signal 1) and activation (signal 2) [7], we did not observe IL‐1β induction by SM‐102 LNPs alone in vivo. Consistent with this, LNPs did not induce IL‐1β secretion or activate NF‐κB in a TLR4 reporter assay (Figure S2B), indicating that LNPs primarily function as signal 2 rather than signal 1 in immune cells. These results align with studies demonstrating that LNPs promote inflammasome activation through reactive oxygen species (ROS) generation and endosomal disruption, and that they exacerbate pre‐existing inflammatory states in vivo [21, 34].

As expected, IP‐10 secretion was induced by both interferon beta (IFNβ) and IFNγ stimulation and was further amplified 3–4‐fold by all LNP formulations, with IFNγ eliciting the strongest responses (Figure 2E). No significant differences were observed between LNP types, although inter‐experimental variability was high. Notably, Alum did not synergize with IFNγ, suggesting that LNPs promote IP‐10 production via a distinct mechanism. Similar synergistic effects were observed with other chemical stressors, including digitonin, thapsigargin, and phorbol 12‐myristate 13‐acetate (PMA) (Figure S2C). IP‐10 has emerged as a key cytokine in innate responses to LNPs and has been linked to vaccine efficacy and, more recently, to adverse events [16, 17, 18, 23]. Importantly, Cao et al. demonstrated that inhibition of the IFNγ/IP‐10 axis ameliorates myocarditis in a preclinical SARS‐CoV‐2 mRNA vaccine model, suggesting that rational modulation of LNP‐driven cytokine programs could mitigate reactogenicity [23].

Using THP‐1 dual‐reporter cells to monitor NF‐κB and interferon regulatory factor (IRF) pathway activation, we further observed synergy between LNPs and both IFNγ and LPS (Figure 2F,G). LNPs and Alum alone induced minimal NF‐κB activity, and IFNs alone did not activate NF‐κB. However, co‐stimulation with LNPs and either IFNγ or LPS resulted in robust NF‐κB activation (Figure 2F).

Taken together, these findings demonstrate that LNPs exhibit only mild pro‐inflammatory activity in vitro under basal conditions but synergize strongly with inflammatory stimuli to induce clinically relevant cytokines, including IL‐1β and IP‐10. Importantly, cytokine responses to LNPs can be categorized into three mechanistically distinct programs: cytotoxicity‐associated responses (e.g. MCP‐1), inflammasome‐mediated responses (IL‐1β), and interferon‐dependent responses (IP‐10 and related chemokines).

### 2.3 LNP Immunogenicity is Strongly Formulation‐Dependent, With PEGylated Lipid Concentration Emerging as a Key Determinant

**FIGURE 3:**
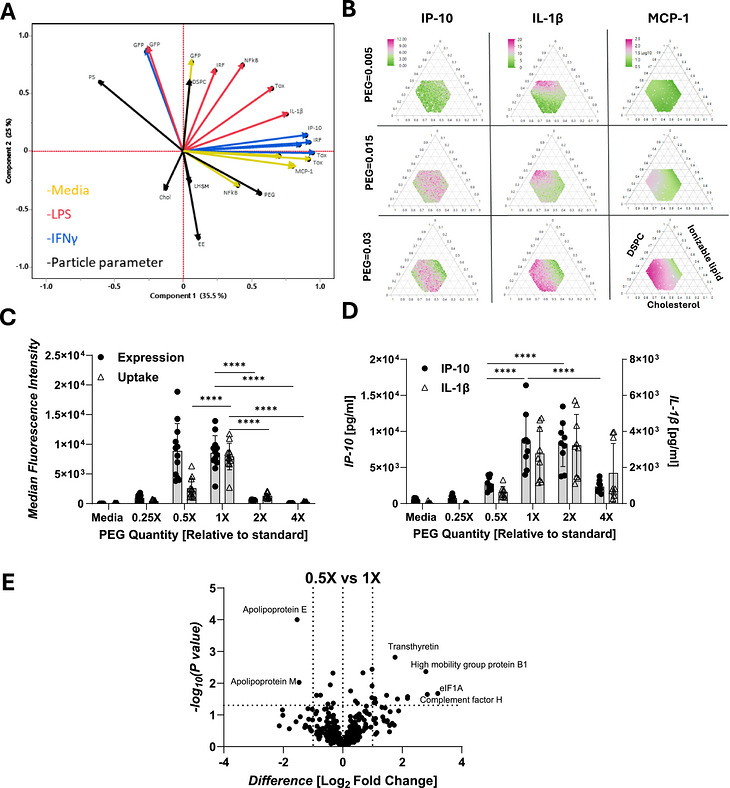
A design of experiments (DoE) approach identifies the contribution of non‐ionizable lipids to immunogenicity. (A) A D‐optimal DoE panel of 15 LNP formulations varying SM‐102, DSPC, cholesterol, and PEG‐DMPE ratios was applied to THP‐1 Dual cells pretreated with LPS, IFNγ, or media. Parameters measured included GFP expression, IRF, NF‐κB, IL‐1β, IP‐10, toxicity, particle size (PS), and polydispersity index (PDI). Principal component analysis (PCA) loading plots were used to visualize relationships among formulation variables, particle properties, and biological readouts. Colors indicate the pre‐stimulation condition associated with each readout (red, LPS; blue, IFNγ; green, and media), while black vectors denote formulation and particle parameters. Vectors pointing in similar directions indicate positive association, whereas near‐perpendicular vectors indicate weaker relationships. (B) Ternary plots illustrate the relationship between LNP composition and cytokine responses (IP‐10, IL‐1β, and MCP‐1) at fixed PEG‐DMPE levels of 0.5%, 1.5%, and 3 mol%. Within each ternary plot, each side of the triangle corresponds to the mol% of one formulation component: ionizable lipid, DSPC, or cholesterol. The composition of any formulation is defined by the intersection of the three grid lines passing through a given point, which indicates the relative proportion of each component. In this way, every location within the triangle represents one unique formulation composition. The color scale indicates the predicted cytokine response across the formulation space, with pink denoting higher and green denoting lower cytokine production. For example, lower IP‐10 responses are predicted across a broader range of formulations at 0.5 mol% PEG, whereas higher IP‐10 responses are more prevalent at 1.5 mol% PEG. (C) LNPs formulated with GFP‐encoding mRNA and Cy5‐labeled GFP‐encoding mRNA at an 80:20 ratio and incremental PEG levels were assessed for cellular uptake (Cy5) and GFP expression in THP‐1 cells by flow cytometry. Bars represent mean ± SD; *n* = 4 independent experiments; individual data points represent technical replicates pooled across experiments. Data were analyzed using two‐way ANOVA. ^****^ *p* < 0.0001. (D) Cells pre‐stimulated with IFNγ or LPS prior to LNP addition were assayed for IP‐10 and IL‐1β production after 48 h. Bars represent mean ± SD; *n* = 3 independent experiments; individual data points represent technical replicates pooled across experiments. Data were analyzed using two‐way ANOVA. ^****^ *p* < 0.0001. (E) Protein corona composition was compared between LNPs containing 0.5x vs. 1x PEG. Data were analyzed using multiple unpaired *t*‐tests without correction for multiple comparisons; *n* = 5 independent batches. ^****^ *p* < 0.0001.

We hypothesized that multiple lipid components contribute to LNP‐driven inflammation and applied a THP‐1 model with IFNγ or LPS preconditioning for DoE screening. Using a DoE matrix, we generated 15 SM‐102 LNP formulations with varying compositions (Table S1) and evaluated them for both immunogenicity and GFP expression. Immunogenicity was assessed under several conditions: media only (monitoring MCP‐1), LPS and IFNγ pre‐stimulation (assessing IL‐1β and IP‐10, respectively), together with readouts including GFP expression, toxicity, IRF, and NF‐κB activity. LNP particle characteristics, including size, PDI, and encapsulation efficiency, were also measured (Table S1). Relationships between parameters were visualized using principal component analysis (PCA) loading plots (Figure 3A), which showed GFP expression aligning strongly with PC2, whereas immunogenic readouts (IP‐10, IL‐1β, and MCP‐1) clustered with PC1, further supporting the conclusion that protein expression is largely independent of immunogenicity in this model. Consistent with prior data, MCP‐1 release correlated closely with cytotoxicity.

Notably, the lipid components SM‐102, cholesterol, and DSPC did not strongly drive immunogenicity, as their vectors were nearly perpendicular to the immunogenic readouts (Figure 3A). Among particle parameters, polyethylene glycol (PEG) concentration was most positively associated with PC1 and immunogenicity. Ternary plots for PEG at 0.5%, 1.5%, and 3% showed that IP‐10 release was strongly PEG‐dependent, with levels rising sharply above 0.5%, largely independent of the other formulation variables (Figure 3B). In contrast, IL‐1β and MCP‐1 responses showed a more complex dependence on formulation composition. While some patterns were most evident at lower PEG content, these responses were not determined by low‐PEG conditions alone and instead reflected contributions from PEG, DSPC, SM‐102, and cholesterol.

To further investigate PEG's role, five LNP formulations spanning 0.25x–4x the standard PEG content (1x = 1.5 mol%) were assessed for uptake, expression, and immunogenicity (Figure 3C,D). Particle size decreased with increasing PEG content, consistent with our previous study [35]. However, dynamic light scattering (DLS) did not indicate particle aggregation across these formulations (Table S1), and Raman spectroscopy did not provide evidence for clustering of the LNP PEG molecules (Figure S3A) [36]. In addition, reducing PEG content from 1x to 0.5x did not substantially alter zeta potential (−1.99 ± 0.46 mV vs. −1.83 ± 1.29 mV, respectively; *n* = 3), suggesting that the differences observed between these formulations are unlikely to be explained by a major change in surface charge alone. At 0.25x PEG, uptake and expression were minimal, increasing at 0.5x and peaking at 1x. Higher PEG levels (2x and 4x) sharply reduced both uptake and expression. Immunogenicity analysis revealed that 1x PEG LNPs induced the highest IP‐10 and IL‐1β responses following IFNγ or LPS stimulation, while 2x PEG LNPs elicited comparable cytokine levels despite low uptake, suggesting uptake‐independent mechanisms. The 0.5x PEG LNPs, with intermediate uptake, were only weakly immunogenic. Comparison of PEG lipids demonstrated that the C14 PEG lipids 1,2‐dimyristoyl‐rac‐glycero‐3‐methoxypolyethylene glycol (PEG‐DMG) and 1,2‐dimyristoyl‐sn‐glycero‐3‐phosphoethanolamine‐N‐[methoxy(polyethylene glycol)‐2000] (PEG‐DMPE) produced similar effects, whereas the non‐shedding C18 PEG lipid 1,2‐distearoyl‐rac‐glycero‐3‐methoxypolyethylene glycol (PEG‐DSG) did not synergize with IFNγ or LPS at any concentration (Figure S3D,E). Direct addition of unformulated PEG lipids failed to induce cytokine responses, confirming that LNP context is required (Figure S3F,G).

To identify potential PEG‐dependent surface ligands that could influence LNP immunogenicity, we characterized the LNP protein corona formed in media supplemented with 10% fetal calf serum (FCS) (Table S2). LNPs were incubated with culture media and subsequently isolated using magnetic beads conjugated to anti‐PEG antibodies. As shown in Figure 3E, the overall protein coronas of LNPs formulated with 0.5x and 1x PEG were broadly similar. However, the 0.5x PEG LNPs exhibited reduced apolipoprotein E (ApoE) abundance in the corona (Figure 3E and Table S2).

Overall, DoE analysis revealed that IP‐10 secretion depends on both ionizable and PEGylated lipid components. This aligns with previous reports showing that IFNγ‐enhanced IP‐10 release is potentiated by NF‐κB‐activating stimuli [37, 38, 39], supporting a model in which LNP‐induced cellular stress amplifies interferon‐dependent signaling [40]. Galectin‐9‐mediated endosomal repair, previously described in non‐immune cells, may represent one such stress‐associated pathway [25]. PEG lipids influence opsonization, biodistribution, cellular uptake, and endosomal trafficking, and have been directly implicated in modulating both LNP immunogenicity and distribution [2, 12, 25, 26, 27, 36, 41, 42]. Mechanistically, our data suggest that the observed differences in IP‐10 induction are not simply attributable to gross particle aggregation or major changes in surface charge and more likely reflect PEG‐dependent changes in LNP properties and their cellular interactions. We and others have shown that initial LNP binding to cells is strongly influenced by protein corona composition, including the presence of lipoprotein‐associated proteins [41]. Consistent with this, we observed lower ApoE association with the lower‐PEG formulation, which may contribute to the reduced cellular uptake observed in our assay and is in line with reports of altered biodistribution for LNPs with reduced PEG content [12]. We also cannot exclude that PEG content influences subsequent internalization and intracellular processing of LNPs, consistent with our previous study linking LNP size to internalization efficiency [35]. Further studies will be required to define these mechanisms more clearly. Importantly, given the established association between elevated IFNγ/IP‐10 signatures, vaccine efficacy, and myocarditis risk [14, 16, 17, 18, 19, 20, 23], rational exploration of LNP formulation space may help attenuate reactogenicity.

### 2.4 LNP‐Mediated mRNA Expression, rather than Innate Immunogenicity, is the Strongest Correlate of Vaccine Efficacy

Using a DoE model with pre‐conditioned THP‐1 cells, we established that all LNP formulations induced IP‐10 production, with responses strongly influenced by PEG content, whereas MCP‐1 release was largely attributable to cytotoxicity driven by either the ionizable lipid or the overall formulation. To validate these in vitro findings and test our hypothesis in vivo, we compared the immunogenicity of LNPs formulated with MC3, SM‐102, LP01, and a DoE‐predicted low‐IP‐10 variant of SM‐102 (SM‐102/DoE).

**FIGURE 4:**
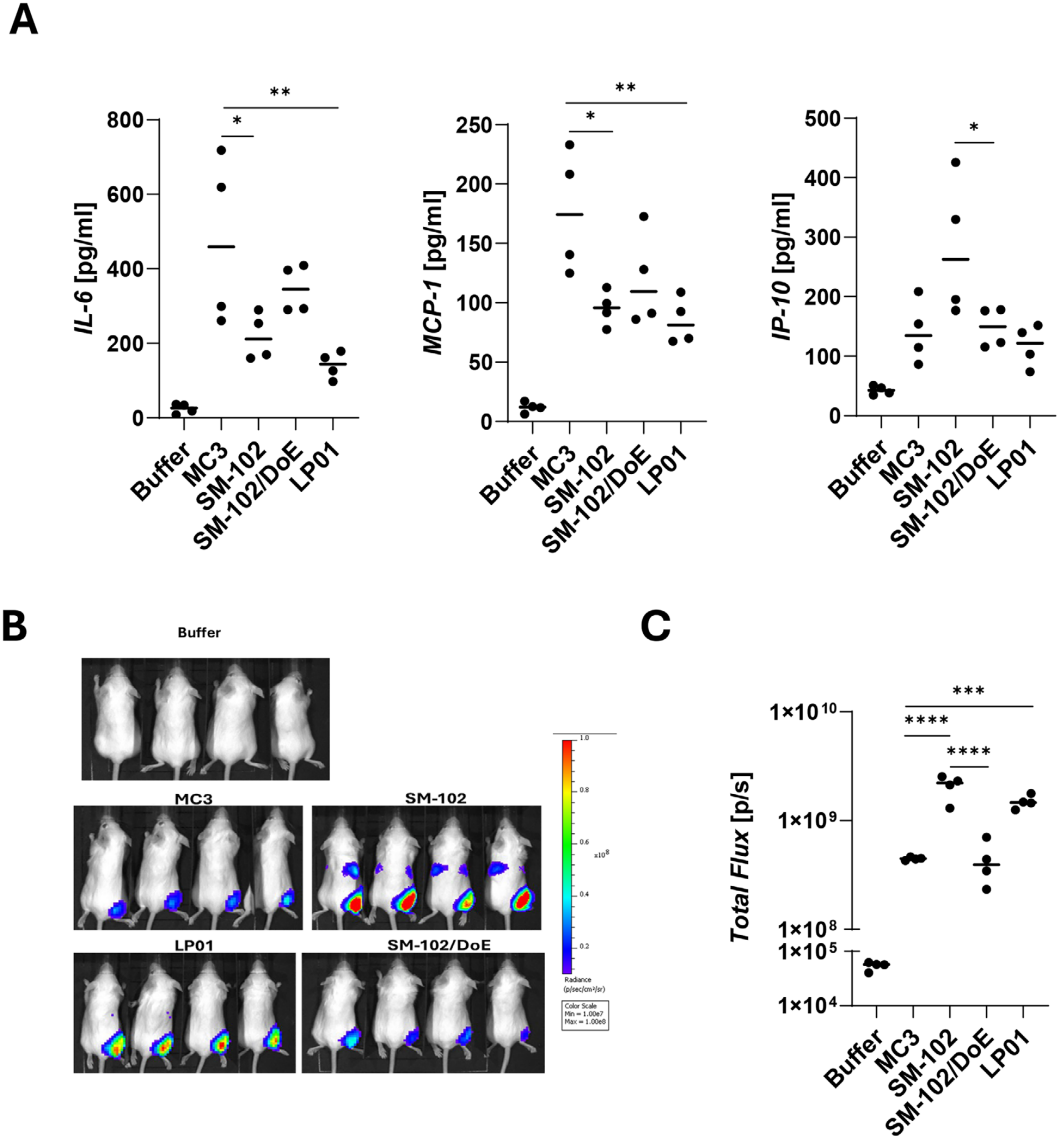
LNP vaccine immunogenicity is driven by the ionizable/PEGylated lipid combination and antigen potency. (A) BALB/c mice (*n* = 4 per group) were injected intramuscularly with luciferase‐expressing mRNA (5 µg, 50 µL) formulated in LNPs containing MC3, SM‐102, or LP01 as the ionizable lipid. A buffer control, as well as a predicted low‐immunogenicity LNP formulation (SM‐102/DoE), were included. Serum was collected 6 h post‐injection, and cytokines were quantified by MSD multiplex ELISA. Individual data points represent individual mice. Data were analyzed using one‐way ANOVA.^*^p < 0.05, ^**^ *p* < 0.01. (B) Luciferase expression was quantified by injection of D‐luciferin (100 µL, 15 mg/mL) 10 min before imaging using an IVIS system. Representative images show luciferase signal at the injection site; regions of interest (ROIs) were drawn manually to quantify local photon flux. (C) Quantification of luciferase expression is shown as total photon flux per mouse (C). Individual data points represent individual mice. Data were analyzed using one‐way ANOVA. ^***^ *p* < 0.001, ^****^ *p* < 0.0001.

LNPs with differing predicted immunogenicity were evaluated in BALB/c mice using luciferase‐encoding mRNA, enabling simultaneous assessment of protein expression and innate immune activation. Serum cytokines were measured 6 h after intramuscular injection using a 6‐plex panel (IL‐1β, IL‐6, interferon alpha (IFNα), IFNγ, MCP‐1, and IP‐10). As shown in Figure 4A, MC3 and SM‐102/DoE elicited the highest IL‐6 responses, whereas SM‐102 and LP01 induced lower levels. MCP‐1 was increased across all LNP formulations and broadly tracked with IL‐6; however, only MC3 was significantly elevated relative to control. IP‐10 was upregulated in all groups, but only SM‐102 showed a statistically significant increase compared with control. Consistent with the in vitro findings, reducing PEGylated lipid content in SM‐102/DoE resulted in decreased IP‐10 induction. IFNγ, IL‐1β, and IFNα remained below the assay's limit of detection.

Gene delivery efficiency was assessed via luciferase activity at 6 h post‐injection. MC3 and SM‐102/DoE exhibited the lowest levels of expression, whereas SM‐102 and LP01 achieved significantly higher signals, with SM‐102 showing the strongest expression. Notably, luciferase signal was detected distal to the injection site in the SM‐102 group, consistent with potential liver expression (Figure 4B,C).

**FIGURE 5:**
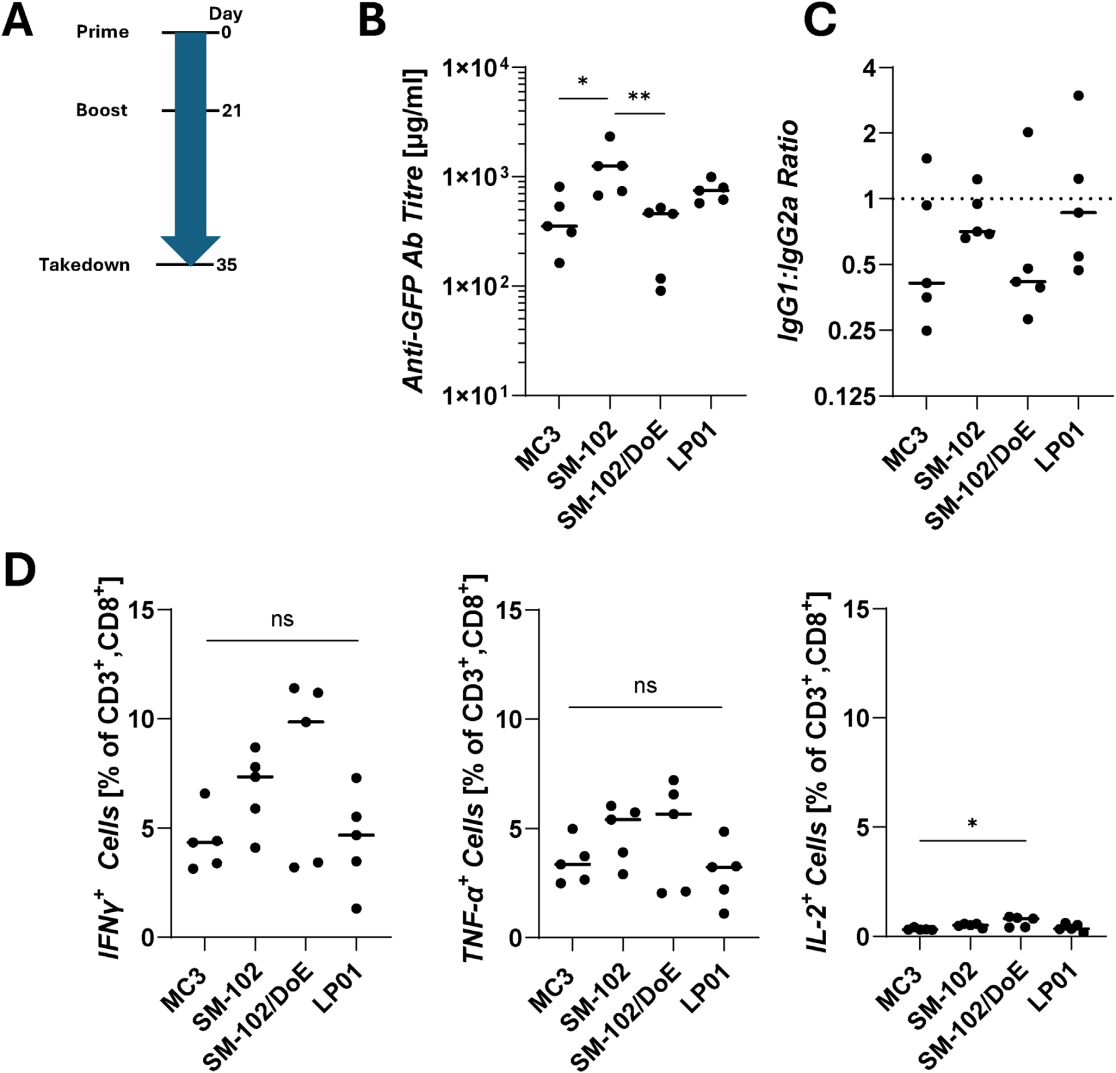
All LNPs elicit robust adaptive responses. (A) Schematic of the vaccination protocol: BALB/c mice (*n* = 5) received intramuscular injections of GFP mRNA LNPs formulated with MC3, SM‐102, or LP01 on days 0 and 21. Tissues were collected on day 35. (B) Serum anti‐GFP IgG titers at day 35 following BALB/c vaccination were quantified by ELISA using anti‐mouse IgG‐HRP and expressed by interpolation from a monoclonal anti‐GFP standard curve. Individual data points represent individual mice. Data were analyzed using one‐way ANOVA. (C) Serum IgG1 and IgG2a isotypes were assessed by ELISA; IgG1/IgG2a signal ratios are presented. Individual data points represent individual mice. (D) Spleens were processed into single‐cell suspensions; splenocytes were stimulated with media or GFP peptide (5 µg/mL) in the presence of brefeldin A, stained for viability, CD3, CD4, and CD8, and then stained intracellularly for IFNγ, TNF‐α, and IL‐2. Flow cytometry was used to identify cytokine‐positive CD8+ T cells. Individual data points represent single mice, and the median is shown. Data were analyzed using Student's *t*‐test. ^*^ *p* < 0.05, ^**^ *p* < 0.01; ns, not significant.

To examine how early innate responses influenced adaptive immunity, GFP was used as the vaccine antigen for continuity with prior experiments. GFP is weakly immunogenic as an unadjuvanted soluble protein but contains a mapped CD8 T‐cell epitope, enabling evaluation of cytotoxic responses [30, 31]. Mice received a two‐dose prime–boost regimen (Figure 5A). No overt changes in mouse behavior or body weight were observed following LNP administration during the study period, although formal toxicology assessments were not performed. Despite differences in innate immunogenicity and expression, all LNPs elicited robust anti‐GFP antibody responses. SM‐102 and LP01 generated higher antibody titers than MC3 and SM‐102/DoE (median endpoint titers: MC3 = 354 µg/mL; SM‐102 = 1,252 µg/mL; LP01 = 749 µg/mL; and SM‐102/DoE = 459 µg/mL) (Figure 5B). SM‐102/DoE showed a significant reduction relative to its parent SM‐102 formulation, whereas differences compared with other lipids were not statistically significant. SM‐102 and LP01 elicited balanced IgG1:IgG2a responses, while MC3 and SM‐102/DoE showed a non‐significant IgG1 bias (Figure 5C).

Cellular immune responses were assessed by ex vivo stimulation of splenocytes with GFP peptide, followed by intracellular cytokine staining and flow cytometric analysis. Antigen‐specific IFNγ–producing CD8 T cells were detected in all groups (∼1%–9%), with no significant differences observed between formulations for IFNγ or TNF‐α responses. A statistically significant difference was observed between SM‐102/DoE and MC3 for IL‐2 production; however, responses were minimal across all groups (<1% of CD8 T cells) (Figure 5D).

**FIGURE 6:**
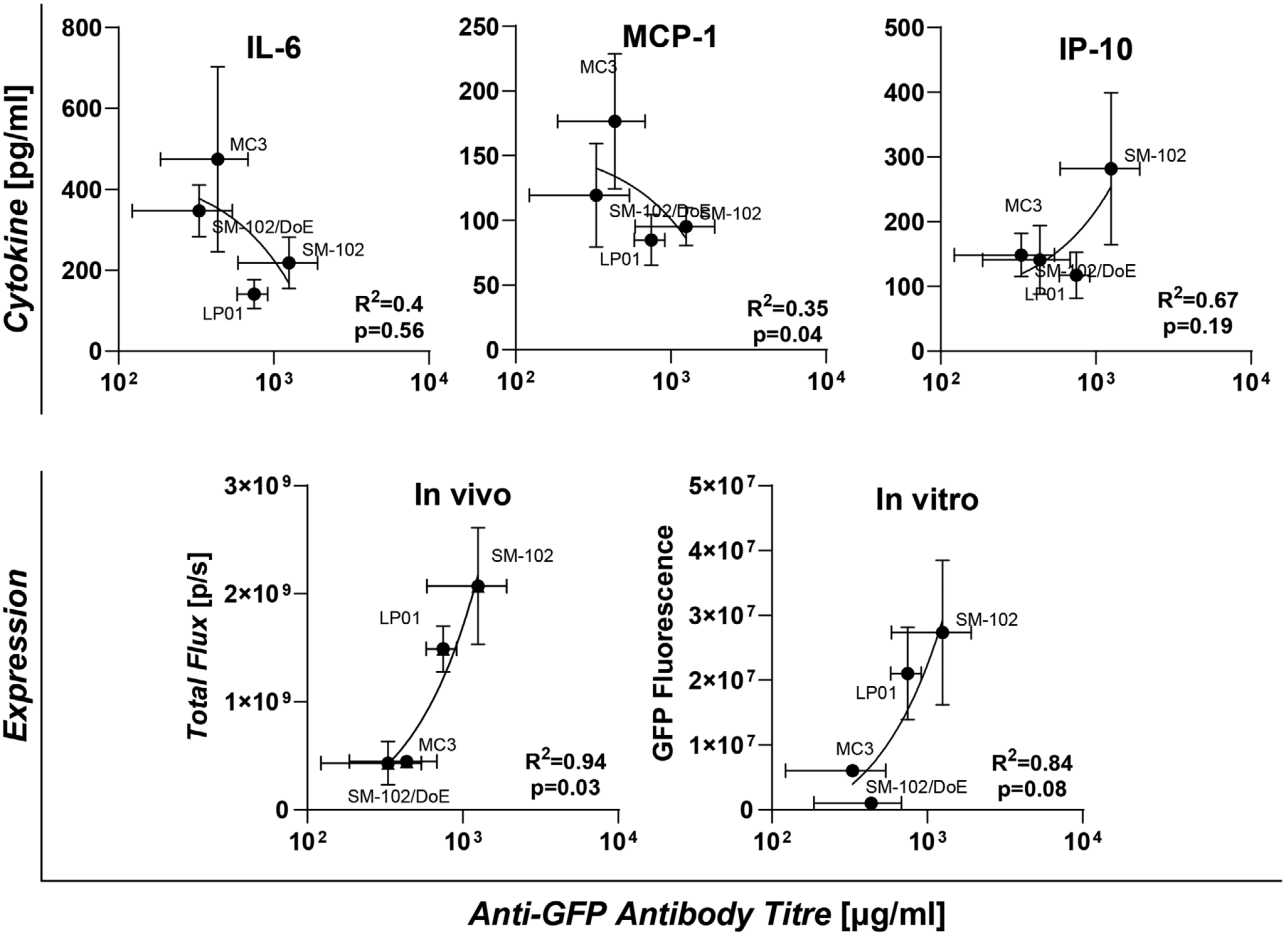
Protein expression in vivo and in vitro shows the strongest correlation with antibody responses. In vivo cytokine data from Figure 4A, in vivo protein expression data from Figure 4C, and in vitro protein expression data from Figure 1C were plotted against serum anti‐GFP antibody titers from Figure 5B. Linear regression was performed using GraphPad Prism software, and the fitted trendline is shown. Error bars represent standard deviation, and R^2^ values are inset.

To identify potential correlates of immunogenicity, innate cytokine responses were plotted against antibody titers. Pro‐inflammatory cytokines IL‐6 and MCP‐1 exhibited weak inverse correlations with antibody titer (R^2^ = 0.40 and 0.35, respectively), whereas IP‐10 showed a stronger positive correlation (R^2^ = 0.67) (Figure 6). These relationships should be interpreted cautiously given the limited dataset, although they may be strengthened with a larger dataset. For example, LP01 elicited relatively strong antibody responses despite a lower IP‐10 signal than SM‐102, indicating that IP‐10 alone is unlikely to fully explain adaptive immunogenicity across formulations. One possible explanation is that cytokine responses must exceed a threshold to support effective antibody induction, beyond which further inflammatory signaling provides no additional benefit. Moreover, the IFNγ/IP‐10 axis may represent a pathway in which efficacy‐associated innate signatures intersect with reactogenicity‐associated biology, including pathways implicated in myocarditis, underscoring the need for rational tuning of LNP composition rather than maximization of innate stimulation [23]. Further studies in appropriate preclinical models are warranted to determine whether formulations with lower IP‐10 induction are associated with reduced myocarditis‐related pathology.

Protein expression demonstrated the strongest correlation with immunogenicity, both in vivo and in vitro (R^2^ = 0.94 and 0.84, respectively) (Figure 6). Across both murine and in vitro models, transgene expression emerged as the most robust predictor of vaccine efficacy, correlating positively with antibody responses. Although statistically significant differences were observed between formulations, these effects were modest, suggesting that baseline LNP immunogenicity is sufficient to support potent immune responses, likely driven by efficient antigen delivery to draining lymph nodes [43]. However, the limited lipid panel examined and the exclusive focus on intramuscular administration restrict broader extrapolation. Future studies should therefore expand lipid and payload diversity and explore alternative routes of administration to further validate PEG‐ionizable lipid interactions.

It should be noted that this analysis was performed using a limited dataset, and the conclusions should be considered preliminary, serving as a foundation for future investigations employing larger panels of LNP formulations. Additionally, the potential contribution of the expressed antigen itself may have been overlooked, as in vivo innate immunogenicity and expression studies were conducted using luciferase, whereas adaptive immune responses were measured against GFP. We and others have recently shown that the type and level of PEG influence LNP biodistribution [12, 36]. Accordingly, future studies incorporating ex vivo tissue analysis, biodistribution measurements, and assessment of local tissue inflammatory responses will be important to determine whether the formulation‐dependent differences in in vivo expression reflect local retention, lymphatic trafficking, or off‐target organ accumulation. Finally, our IFNγ‐preconditioned in vitro model also represents a deliberately sensitized inflammatory context used to reveal the contribution of amplified interferon‐responsive pathways to LNP immunogenicity. The resulting IP‐10 responses should therefore not be interpreted as direct quantitative surrogates of in vivo cytokine magnitude. Further studies will be required to clarify the cross‐talk between physiological sources of interferon, including T cells and NK cells, and myeloid cells producing IP‐10.

## 3 Conclusion

This study demonstrates that LNPs elicit distinct cytokine programs governed by the interplay between ionizable and PEGylated lipid components. Using a DoE framework, we identify PEG‐ionizable lipid pairing as a major determinant of interferon‐linked chemokine responses and highlight in vivo transgene expression, rather than innate cytokine magnitude, as the strongest predictor of adaptive immunity. Together, these findings establish a pragmatic, materials‐driven framework to guide rational LNP design and early screening of safer vaccine candidates.

## 4 Materials and Methods

### 4.1 Materials

1,2‐dimyristoyl‐sn‐glycero‐3‐phosphoethanolamine‐N‐[methoxy(polyethylene glycol)‐2000] (PEG‐DMPE) was obtained from NOF America Corporation, 1,2‐dimyristoyl‐rac‐glycero‐3‐methoxypolyethylene glycol‐2000 (PEG‐DMG) and 1,2‐distearoyl‐rac‐glycero‐3‐methoxypolyethylene glycol‐2000 (PEG‐DSG) were purchased from Avanti Polar Lipids, distearoylphosphatidylcholine (DSPC), cholesterol, bovine serum albumin (BSA), and ethanol were purchased from Sigma. Clean Cap 5‐methoxyuridine‐modified mRNA encoding enhanced GFP, firefly luciferase, as well as Cy‐5‐labeled mRNA encoding enhanced GFP were obtained from TriLink. Fluorophore conjugated anti mouse monoclonal antibodies targeting CD3 PerCP‐Cy5.5 (145‐2C11), CD8 BV650 (53‐6.7), CD4 BV605 (RM4‐5), IFNγ PE (XMG1.2), IL‐2 APC (JES6‐5H4), and TNF‐α BV785 (MP6‐XT22) were purchased from Biolegend as was 3,3′,5,5′‐tetramethylbenzidine (TMB) substrate solution, enzyme‐linked immunosorbent assay (ELISA) stop solution, brefeldin A, Zombie UV/Aqua, intracellular staining buffer, red blood cell lysis buffer, cell stimulation cocktail (PMA + Ionomycin), and mouse anti‐GFP (1GFP63). Duo Set ELISA kits specific for human IL‐1β, IP‐10, and MCP‐1 were purchased from R&D Systems. Customized Meso Scale Discovery (MSD) U‐plex kits were purchased from Meso Scale Discovery. Recombinant cytokines were all from Peprotec. The bioconjugatable LPS‐EK, Quanti‐Blue/ Quanti‐Luc reagents were supplied by Invivogen. Quant‐it RiboGreen, Indo‐1 AM dye, and Goat anti‐mouse IgG horseradish peroxidase (HRP) conjugate were provided by Invitrogen. Cell Titre Glo, One‐Glo luciferase assay system, and 3/7 Caspase Glo were from Promega. All tissue culture reagents, unless stated, were from Gibco. Additional chemical reagents were purchased from Sigma. Enhanced GFP peptide (200‐208, HYLSTQSAL) was from AnaSpec (ANA65302). Recombinant Enhanced Green Fluorescent Protein (EGFP) from Cambridge Bioscience (#230‐30040‐10). Goat anti‐Mouse IgG HRP was from ThermoFisher (A16078), goat anti‐mouse IgG1:HRP (STAR132P), and goat anti‐mouse IgG2a:HRP (STAR133P) were from Bio‐Rad. Reagent‐grade formic acid (Sigma‐Aldrich), and high‐performance liquid chromatography (HPLC)‐grade isopropanol, acetonitrile, and water (Fisher Scientific) were used for both extraction and liquid chromatography‐tandem mass spectrometry (LC‐MS/MS) solvents. Mitochondria Isolation kit for cultured cells was obtained from Thermo Scientific (#89874).

### 4.2 Cells and Cell Culture

THP‐1 dual monocytic cells (THP‐1) were obtained from Invivogen. These cells express luciferase under the control of an interferon‐stimulated gene 54 (ISG54) minimal promoter with five IFN‐stimulated response elements (ISREs). Additionally, they produce secreted embryonic alkaline phosphatase (SEAP) under the control of the IFNβ promoter with an NF‐kB consensus transcriptional response element and three copies of c‐Rel binding site. THP‐1 Dual and THP‐1 nuclear factor of activated T cells (NFAT) luciferase reporter cells were stored and supplied by the AstraZeneca cell bank. All cell lines were confirmed to be mycoplasma‐free. Cells were maintained in RPMI‐1640 + Glutamax (2 mm) with 10% FCS (cRPMI) incubated at 37°C in a humidified incubator with 5% CO_2_. Cells were split at a ratio of 1:10, 2–3 times per week.

### 4.3 Peripheral Blood Mononuclear Cell (PBMC) Isolation

Peripheral blood mononuclear cells (PBMCs) were isolated using density gradient centrifugation with Ficoll‐Paque as follows. Whole blood (approximately 10 mL per sample) collected in cones was decanted into sterile 50 mL conical tubes. The blood volume was adjusted to 40 mL with Dulbecco's phosphate‐buffered saline (PBS). For density gradient separation, Ficoll‐Paque (15 mL) was added to a 50 mL tube, and diluted blood (20 mL) was carefully layered on top, ensuring not to disturb the interface. The tubes were centrifuged at 400×g for 40 min at room temperature using safety lids and with the brake turned off. After centrifugation, the clear plasma layer was aspirated. The PBMC layer (buffy coat) located at the interface of plasma and Ficoll‐Paque was carefully collected and transferred to a new 50 mL conical tube. The collected cells were brought to a final volume of 40 mL with PBS and pelleted by centrifugation at 200×g for 10 min. The supernatant was aspirated, and the cell pellet was resuspended in ammonium‐chloride‐potassium (ACK) red blood cell lysis buffer (5–7 mL), followed by incubation for 5 min at room temperature to lyse any residual erythrocytes. After lysis, PBS was added to a final volume of 40 mL, and samples were centrifuged again at 200×g for 10 min. Finally, the supernatant was carefully removed, and the cell pellet was resuspended in an appropriate buffer for downstream applications. Cell concentration and viability were determined using trypan blue exclusion with a hemocytometer.

### 4.4 Animals and In Vivo Studies

All animal studies were performed by personal licenses covered by the United Kingdom (UK) Home Office project licence (PP8950634) and in accordance with the UK Animals (Scientific Procedures) Act 1986. Animals were housed at King's College London and experiments were approved by the King's College London Animal Welfare and Ethical Review Body and the UK Home Office. BALB/c or C57BL/6 mice, 4–6 weeks old (Envigo) were used as specified.

### 4.5 LNP Formulation and Characterization

LNPs were produced as previously described [41]. In brief, an ethanolic solution of ionizable lipid, DSPC, cholesterol, and PEGylated lipid at a 50:10:38.5:1.5 molar ratio was rapidly mixed with an aqueous solution (Citrate buffer, pH 3) of RNA using a NanoAssemblr Ignite (Precision Nanosystems). The nitrogen‐to‐phosphate ratio (N:P) ratio was maintained at six for all formulations. Ethanol was removed by dialysis overnight against PBS with a Slide‐A‐lyzer dialysis cartridge (10,000 molecular weight cut‐off (MWCO), Thermofisher). LNPs were collected, sterilized by filtration through a 0.22 µm syringe‐driven filter, and stored at 4°C. For long‐term storage, LNPs were dialyzed against 20 mm Tris buffer (pH 7.4), supplemented with 8% (w/v) sucrose, and then frozen, as previously described [27]. Where LNP distribution analysis was to be performed, Cy5‐labeled GFP‐encoding mRNA was included at 20% (w/w) of total RNA. RNA loading was determined by the RiboGreen assay. Diluted LNPs (50 µl) were incubated with either Tris‐EDTA (TE) buffer or TE buffer with 2%Triton X‐100 (50 µL) for 10 min at 37°C in a black 96‐well plate (Nunc). Following incubation TE buffer containing Quant‐it RiboGreen diluted 1:100 (100 µL) was added to each well. Plates were read immediately and fluorescence signal at 480/520 nm was determined using an EnVision multilabel reader (Perkin Elmer). RNA content was determined from interpolation of the signal from a standard curve of corresponding RNA. Encapsulation efficiency was calculated according to Equation (1):

(1) $$\begin{array}{cc} & \text{Encapsulation efficiency}\left(\%\right)\\ & =\left(1-\frac{\text{mRNA quantity without triton X}}{\text{mRNA quantity with triton X}}\right)\times 100 \end{array}$$

Particle size and polydispersity index (PDI) were measured using a ZetaSizer Ultra (Malvern). In brief LNPs (10 µL) were diluted in 1 mL of PBS and loaded into a cuvette. Zeta potential was measured for selected formulations using disposable capillary cells after dilution in phosphate buffer solution (10 mM phosphate, pH 7.2), following the manufacturer's instructions. Three replicate measurements were recorded for these formulations, and all data met quality control criteria.

### 4.6 Single‑Particle Automated Raman Trapping Analysis

Raman characterization at the single‐particle level was conducted on the single‐particle automated Raman trapping analysis (SPARTA) platform using 785 nm excitation, as previously described [36]. Prior to acquisition, LNP dispersions were freshly diluted in PBS. For each sample replicate (*n* = 3), the system was configured for 200 automated trapping attempts. Raman spectra were recorded only from particles that were successfully trapped, using an acquisition time of 10 s per particle. All spectra were processed in SPARTA Discovery (version 1.2.1). Buffer‐related signal was reduced by subtracting 95% of an averaged PBS reference spectrum from each single‐particle spectrum. Spectra were subsequently limited to 600–1800 cm^−^^1^, corresponding to the principal fingerprint region used for interpretation. Background removal was performed with a Whittaker baseline correction algorithm configured with log smoothness 7 and differential order 2. Spectral smoothing was then applied using a second‐order Savitzky‐Golay filter. For inter‐particle comparison, spectra were normalized to unit total area (area under the curve (AUC) = 1).

### 4.7 Cell Stimulation With LNP

THP‐1 Dual cells were plated in a 96‐well flat clear‐bottom black plate (Corning) at a density of 50 000 cells per well in cRPMI. Where specified, cells were stimulated with either LPS at a final concentration of 1 µg/mL, recombinant IFNγ at a final concentration of 20 ng/mL or left unstimulated. Cells were incubated for 1 or 4 h for interferon or LPS, respectively. Following incubation, LNPs with various cargos, diluted in cRPMI, were added to a final concentration of 1 µg/ml. Stimuli were incubated for 48 h as described. Where GFP protein expression was analyzed, plates were incubated in an Incucyte S5 (Sartorius), and five images were obtained for each well with a 10x objective lens and a GFP filter set at a set time interval. Images were analyzed using Incucyte Rev 2 software (Sartorius), and data is plotted as the average total integrated fluorescence intensity of ≥4 images per well.

Supernatant obtained from stimulation was either tested immediately or frozen at −70°C for later testing. Cell viability and caspase activity following treatment were immediately tested using CellTitre‐Glo and Caspase‐Glo 3/7, respectively, according to the manufacturer's instructions. All measurements were made on a SpectraMax iD5 (Molecular Devices). Cell viability is plotted as a percentage of viable cells by comparing the signal obtained from treated cells with the signal obtained from untreated cells. Caspase activity is presented as a fold change by dividing the treated cell signal by the untreated cell signal.

### 4.8 Evaluation of THP‐1 Dual IRF and NF‐kB Reporters

Supernatant from THP‐1 Dual cell stimulations was added to either a clear 96‐well MaxiSorp ELISA plate (Nunc) (20 µL) or a 96‐well opaque white plate (Corning) (10 µl) for NF‐kB or IRF reporter assays, respectively. NF‐kB activation was determined by adding 180 µL of diluted Quanti‐Blue to each well and incubating for 45 min at 37°C, absorbance was read at OD_620_. IRF activity was measured by adding hydrated Quanti‐Luc (50 µL) to each well. All measurements were made on an EnVision Multilabel plate reader (PerkinElmer) or a SpectraMax iD5 (Molecular Devices)

### 4.9 Cytokine ELISA and MSD Analysis of Supernatant

Single cytokine analysis was carried out using Duo Set ELISA kits performed according to the manufacturer's instructions. ELISA MaxiSorp ELISA plates (Nunc) were coated overnight with capture antibody diluted to the appropriate concentration in PBS. Plates were washed four times with PBS + Tween 20 0.05% (v/v) (Wash buffer) by adding 200 µL then removing and blocking with PBS supplemented with 1% BSA (w/v) (block buffer) (200 µL) for 1 h followed by washing as described. Cell culture supernatants were diluted by a preoptimized dilution factor in block buffer and 100 µL was added to the relevant well of the plate. Plates were incubated with shaking for 2 h at room temperature before being washed as described. Biotinylated detection antibody (100 µL), diluted to the specified concentration in block buffer, was added to each well and incubated for 2 h. Plates were washed as described above and horseradish peroxidase (HRP)‐conjugated streptavidin (100 µL) was added to each well and incubated for 45 min before washing as described. Plates were developed by the addition of 3,3′,5,5′‐tetramethylbenzidine substrate (TMB) (100 µL) and incubated for 20 min, color development was stopped with the Stop solution (50 µL). Absorbance at OD_450nm_, and OD_570nm_ were read on an EnVision Multilabel plate reader (Perkin Elmer) or a SpectraMax iD5 (Molecular Devices). To determine analyte levels the OD_570nm_ value was subtracted from the OD_450nm_ value to control for nonspecific background absorbance. A standard curve was then obtained by plotting the absorbance against the concentration and performing a 4‐parameter fit analysis. Cytokine concentration was calculated by interpolation from the standard curve, followed by correction for the dilution factor.

Multiplexed cytokine analysis was performed with a custom MSD U‐plex kit (Meso Scale Discovery) in accordance with the manufacturer's instructions. In summary, plates were prepared by reacting biotin‐conjugated anti‐cytokine antibody (200 µL) with a unique linker (300 µL) for 30 min. The reaction was stopped by the addition of excess stop buffer (200 µL) and incubation for a further 30 min. Pooled capture antibodies (50 µL) were added to each well of an MSD plate followed by incubation for 1 h at room temperature with shaking. Plates were washed as described for standard ELISA. Cell culture supernatant was diluted with block buffer and added to wells of an MSD plate followed by incubation for 2 h at room temperature with shaking. Plates were washed as described above and 50 µL of Sulfo‐Tag‐conjugated detection antibody, diluted in the specified buffer, was added. Plates were incubated for 1 h and washed as described above. Read buffer (150 µL) was added and the plates were read on a Meso Sector S 600 plate reader (Meso Scale Discovery). Analysis was performed using Discovery Workbench software (Meso Scale Discovery).

### 4.10 Flow Cytometry

Staining for flow cytometry was performed as follows: cells were harvested by centrifugation at 1500 rpm for 3 min at 4°C. Pellets were resuspended in PBS (50 µL) and stained with Zombie UV/Aqua, diluted 1:250, in PBS (50 µL) for 20 min in the dark at room temperature. The reaction was stopped by the addition of PBS supplemented with 1% bovine serum albumin (BSA) (100 µL). Cells were washed by centrifugation as above followed by the addition of PBS supplemented with 1% BSA (200 µL), repeated twice. Cells were surface‐stained with relevant fluorophore‐conjugated monoclonal antibodies (all diluted 1:200) by adding diluted antibodies and incubating for 20 min at room temperature. Cells were washed as described. Where required, cells were fixed with 4% paraformaldehyde (PFA) for 30 min at room temperature, followed by resuspension in PBS supplemented with 1% BSA.

For intracellular staining, after fixation, cells were washed three times with 1x intracellular staining buffer (200 µL) by centrifuging at 1500 rpm for 5 min. Intracellular target‐specific monoclonal antibodies were diluted (1:200) with intracellular staining buffer and added to cells. Cells were incubated for 30 min at room temperature, then washed three times with intracellular wash buffer as described. Finally, cells were resuspended in PBS supplemented with 1% BSA prior to acquisition on a BD Fortessa (Becton and Dickinson) equipped with five lasers. Data analysis was performed using FlowJo (TreeStar). Cells were first gated on forward/side scatter before the viable cell population was analyzed by gating on Zombie UV/Aqua‐low population.

### 4.11 Design of Experiments (DoE), Modeling, and Simulations

Design of experiments (DoE) approach was employed to investigate the effects of four mixture variables (i.e., LNP lipid components) on LNP immunogenicity and in vitro gene expression. JMP Pro 17 software (SAS Institute Inc.) was used to generate a custom D‐optimal design with 15 runs. The factor ranges were set as follows: ionizable lipid (20%–60%), phospholipid (5%–50%), cholesterol (5%–50%), and PEG (0.5%–3%). Scheffe cubic model terms were specified to create the design. This model is suitable for mixture experiments where a higher‐order polynomial is needed to model the curvature in the response surface. After collecting response data, self‐validated ensemble modeling (SVEM Lasso) was employed to model the nonlinear relationship between LNP components and the measured responses. JMP Pro 17 software (SAS Institute Inc.) was used for model development and analysis. The models were evaluated using various metrics including R^2^ and Root Mean Squared Error (RMSE). The developed models were utilized to interpolate the entire design space, with the predicted values color‐coded to highlight variations in responses relative to LNP composition.

### 4.12 Principal Component Analysis

Principal Component Analysis (PCA) was employed to explore the correlations and relationships between 21 properties of LNPs covering LNP components, physical properties, and various cellular responses using JMP Pro 17 (SAS Institute Inc.). PCA was chosen for dimensionality reduction and exploration of correlations due to its ability to transform the original variables into a set of uncorrelated principal components. These components capture the maximum variance in the data with a minimal number of variables, simplifying the analysis and interpretation. The eigenvalues indicated the amount of variance explained by each principal component, with the first four principal components accounting for >80% of the variation in the dataset.

### 4.13 LNP Corona Isolation

The method of LNP corona isolation has been previously published [41]. In brief, LNPs were exposed to cell culture media containing 10% FCS at a concentration of 4 µg/mL mRNA (equivalent to 200 ng mRNA LNPs dose). For corona isolation, M‐270 Epoxy Dynabeads (Thermo Fisher Scientific, #14321D) were functionalized with monoclonal anti‐PEG antibodies [MABS1963] (Merck) following the manufacturer's protocol. The isolation procedure was conducted using a KingFisher Flex magnetic purifier (Thermo Fisher Scientific) equipped with 96 magnetic rod heads to effectively separate LNP‐corona complexes. The isolation protocol consisted of a 20 min incubation of antibody‐conjugated Dynabeads with LNPs in media using a 1:1 antibody:mRNA ratio (w/w) with gentle agitation. Following incubation, magnetic rods extracted the Dynabeads, which were subsequently washed four times with PBS. LNP‐corona complexes were eluted from the Dynabeads using a basic elution buffer (0.5 m NH_4_OH, 0.5 mm EDTA).

### 4.14 LNP Protein Corona Proteomics Analysis

Corona proteins were recovered from individual plasma samples containing equivalent amounts of mRNA. The optimized protein digestion workflow began with a mixture of 10 mm tris(2‐carboxyethyl)phosphine (TCEP) bond‐breaker solution (Thermo Fisher Scientific, #77720) and 20 mm 2‐chloroacetamide reagent (Merck, #22790) in 50 mm tris(hydroxymethyl)aminomethane (tris) buffer. The mixture and samples were placed on a plate shaker at 90°C and 850 rpm for 10 min (denaturation/reduction/alkylation). Proteins underwent overnight digestion with trypsin (Merck, #EMS0006), with the reaction terminated by the addition of formic acid (1.1%). Peptide analysis was performed on a Q‐Exactive HF mass spectrometer (Thermo Fisher Scientific) coupled to an Evosep One automatic sample loader (Evosep). The system utilized Evotip disposable C18 trap columns for in‐line peptide desalting and purification immediately before analytical separation. Using a preset 60 samples per day (60‐SPD) loading sequence, purified peptides were separated on an 8 cm analytical reverse‐phase column (Evosep) with gradient offset focusing. Mass spectrometry data were analyzed using MaxQuant software (v1.6.6.0). Protein identification was conducted against the Uniprot FASTA database (Bovine UP000009136). N‐terminal acetylation and methionine oxidation were set as variable modifications, while cysteine carbamidomethylation was designated as a fixed modification. The false discovery rate was maintained at 1% for both proteins and peptides (minimum length: seven amino acids) through reverse database searching. Trypsin specificity was defined as cleavage C‐terminal to arginine and lysine residues, allowing a maximum of two missed cleavages. Peptide identification parameters included an initial precursor mass deviation tolerance up to 6 ppm and a main mass deviation tolerance of 20 ppm. The matching between runs feature was enabled across samples, and proteins matching the reversed database were excluded. For protein quantification, MaxQuant calculated raw protein intensities as the sum of all identified peptide intensities. Label‐free quantification (LFQ) and intensity‐based absolute quantification (iBAQ) were derived from raw protein intensities with a minimum peptide ratio count of 1. Statistical comparisons of identified proteins between samples were conducted using Perseus software (Perseus v2.1.5) and Qlucore Omic Explorer (Qlucore v3.9) based on LFQ intensities.

### 4.15 Quantitation of Ionizable Lipids in Cellular Fractions In Vitro

THP‐1 cells (1 × 10^6^ cells per mL, 5 mL total volume) were treated with equimolar doses of either MC3, SM‐102, or LP01 (1 µg/mL) for 24 h. Following incubation, the samples were prepared for analysis using a Mitochondria Isolation kit to measure the biodistribution of the ionizable lipids in mitochondria‐enriched and cytosol fractions. For accurate quantitation, calibration standards were prepared using reference solutions over a precise analytical range (0.05–50 nm). All calibrators and cellular samples (25 µL) were diluted in 2% formic acid in 49:49 acetonitrile:isopropanol (150 µL), the sample plate was mixed for 2 min at 9000 rpm and centrifuged at 3000 x g for 3 min. The supernatant (50 µL) was then diluted again in 0.1% formic acid in 45:25:30 acetonitrile: isopropanol: water (100 µL), and mixed for 5 min at 1200 rpm, prior to analysis.

All analytical procedures were performed on an ACQUITY I‐Class (Waters) LC system coupled to an AB SCIEX 5000 triple quadrupole mass spectrometer (Sciex). The extracted sample was injected onto an Acquity CSH C18 Column (2.1 mm x 50 mm, 1.7 µm, Waters) set at 50°C, with starting mobile phases set to 55% A (0.1% formic acid (40:60 acetonitrile: water)) and 45% B (0.1% formic acid (50:50 acetonitrile: isopropanol)) and a flow rate of 0.5 mL/min. The analytes were separated over a 2 min gradient from 45% to 90% B, followed by a flush of 98% B for 1 min before returning to initial conditions, resulting in an overall runtime of 3 min. The sample manager wash solvent was 50:50 acetonitrile:isopropanol, and the sample purge solvent was 0.1% formic acid (40:40:20 acetonitrile: isopropanol: water). TurboIonSpray ionization was applied in positive mode, within the mass spectrometer source (600°C), with an ionspray voltage of 3 kV, curtain gas of 35 psi, and a high collision gas setting. The chromatographic data were processed using Sciex Analyst (version 1.7.2), lipids were detected using specific LC retention times and Q1/Q3 ion transitions.

### 4.16 Ex Vivo Stimulation of Whole Splenocytes

Spleens were excised from mice and macerated through a 40 µm cell strainer (BD Bioscience). Tissue extract was centrifuged at 1500 rpm, 5 min. Red blood cells in the pellets were lysed by incubation with 1X RBC lysis buffer (5 mL, 5 min). Lysis was stopped by the addition of cRPMI (25 mL), and live cells were counted using trypan blue exclusion. Cells were plated at a density of 750 000 cells per well in a round‐bottom 96‐well plate. Brefeldin A was diluted and added to each well to a final concentration of 1X. To measure antigen‐specific responses, a peptide corresponding to the minimal immunodominant MHC class I epitope of the GFP protein was added to a final concentration of 5 µg/ml. For each mouse an unstimulated well served as a control for background cytokine release. Cells were cultured under standard conditions for 16 h. After incubation wells were harvested, and intracellular cytokine staining was performed as described below.

### 4.17 Intracellular Cytokine Staining (ICS) for T‐Cell Analysis

Splenocytes were isolated from mice on day 35 post‐immunization and stimulated ex vivo for 2 h with EGFP (200‐208) (5 µg/mL) or cell activation cocktail (PMA/ionomycin, 1×) as a positive control, in the presence of brefeldin A (5 µg/mL). Following stimulation, cells were stained for surface markers and intracellular cytokines. Cells were pelleted by centrifugation (1500 rpm, 3 min, 4°C) and resuspended in PBS and stained with Zombie UV/Aqua (1:250) for 20 min in the dark at room temperature. The reaction was stopped by the addition of PBS supplemented with 1% BSA (PBS + 1% BSA), and cells were washed twice in PBS + 1% BSA by centrifugation. Cells were stained with fluorophore‐conjugated monoclonal antibodies (1:200 in PBS + 1% BSA) by incubating for 20 min at room temperature and washed as above. Next, cells were fixed with 4% paraformaldehyde (30 min at room temperature), washed, and resuspended in PBS + 1% BSA. After fixation and permeabilization, cells were stained for intracellular TNF‐α, IL‐4, IFNγ, and IL‐2. Cells were washed three times with intracellular staining buffer by centrifuging at 1 500 rpm for 5 min. Antibodies were diluted (1:200) in intracellular staining buffer and incubated with cells for 30 min at room temperature. Cells were then washed three times with intracellular wash buffer and were resuspended in PBS + 1% BSA. Samples were acquired on a BD Fortessa (Becton Dickinson) and analyzed using FlowJo (Tree Star, Inc.). Cells were first gated on forward/side scatter (FSC/SSC), followed by gating on singlets (FSC‐A vs. FSC‐H) and the viable cell population, identified as the Zombie UV/Aqua‐low population. CD3+ cells were then selected and further gated into CD4+ or CD8+ subsets for cytokine expression analysis.

### 4.18 Statistical Analysis

All quantitative data are presented as mean ± SD or mean ± SEM, as specified in the corresponding figure legends. Exact sample sizes (n) for each experiment are provided in the relevant figure legends and refer to independent biological replicates unless otherwise stated. For animal studies, each individual data point represents one animal. Where technical replicates were included, this is indicated in the corresponding figure legends. No data transformation was applied unless otherwise stated. Percentage and fold‐change values were calculated relative to the relevant control condition as described in the Methods and figure legends. No additional normalization was applied unless explicitly specified for a given assay. No data points were excluded as outliers unless pre‐defined technical quality‐control criteria were not met. For proteomics analyses, preprocessing and quantification were performed as described in the LNP Protein Corona Proteomics Analysis section, including LFQ‐based statistical comparisons.

For comparisons between two groups, unpaired, two‐tailed Student's *t*‐tests were used. For comparisons involving three or more groups, one‐way or two‐way ANOVA was applied, as appropriate, with post hoc multiple‐comparisons correction where applicable, as indicated in the figure legends. Correlation analyses were performed using linear regression, and R^2^ values are reported where applicable. All tests were two‐sided, and differences were considered statistically significant at α = 0.05. Significance levels are denoted as *p* < 0.05, *p* < 0.01, *p* < 0.001, and *p* < 0.0001.

The suitability of the selected statistical tests was considered based on the experimental design, the use of independent biological replicates, and the distribution of the measured data within each experiment. No data points were omitted on the basis of the statistical outcome. Statistical analyses were performed using GraphPad Prism (v10.4.1 (627), Windows 64‐bit), unless otherwise stated. Design of experiments (DoE) modeling and principal component analysis (PCA) were performed using JMP Pro 17 (SAS Institute Inc.). Proteomics statistical analyses were performed using Perseus (v2.1.5) and Qlucore Omics Explorer (v3.9) based on LFQ intensities.

## Funding

This study was funded by the AstraZeneca R&D PostDoc Program.

## Conflicts of Interest

Belal I. Hanafy, Chuan‐En Lu, Rachel E. Foreman, Ramesh Soundararajan, Sara Marelli, Kai Liu, Philippe Collin, Lennart Lindfors, Alan Sabirsh, Mariarosa Mazza, Stephanie M. Bates, George Thom and Alexander N Kapustin are all current employees of AstraZeneca and may hold shares in AstraZeneca.

## Supporting information




**Supporting File**: smll74283‐sup‐0001‐SuppMat.docx.

## Data Availability

All relevant data supporting the findings of this study are included within the manuscript and its Supplementary Information files. Original raw data, including but not limited to mass spectrometry proteomics data, flow cytometry files, and comprehensive raw outputs from the design of experiments (DoE) modeling, are available upon reasonable request from the corresponding author, Alexander Kapustin.
