# Supplementary material for: Defining Composition–Cytokine Relationships Enables the Design of Lipid Nanoparticles with Programmed Immunogenicity
Source: Small. 2026 Jul 20;22(45):e74283. doi: 10.1002/smll.74283 (PMC13472245; doi:10.1002/smll.74283)
Supplement: Supplementary file 1 [file SMLL-22-e74283-s001.docx]

**Supporting Information**

**Defining Composition–Cytokine Relationships Enables the Design of Lipid Nanoparticles with Programmed Immunogenicity**

Adam Alexander Walters^1^, Belal I. Hanafy^1^, Chuan-En Lu^2^, Sara Marelli^3^, Alaa Zam^4^, Kai Liu^2^, Rachel E. Foreman^5^, Ramesh Soundararajan^1^, Philippe Collin^6^, Lennart Lindfors^2^, Alan Sabirsh^2^, Mariarosa Mazza^1^, Khuloud T. Al-Jamal^4^, Stephanie Bates^7^, George Thom^3^, Jan Rehwinkel^8^, Alexander N Kapustin^1^

**Figure S1. In vitro LNP biodegradability reveals slower MC3 degradation.** (**A**) Representative chromatograms and instrument schematic illustrating the quantitation of ionizable lipids by liquid chromatography coupled to mass spectrometry (LC-MS/MS). Lipids were identified by specific LC retention times and Q1/Q3 ion transitions (MC3: 1.33 min, 644.6/132.1; SM-102: 1.16 min, 711.2/472.9; LP01: 1.21 min, 853.7/547.4). (**B**) Concentrations of lipids in cellular fractions were quantified following treatment of THP-1 cells with equimolar doses of MC3-, SM-102-, or LP01-formulated LNPs. Lipid distribution and accumulation varied between LNP formulations, and the data are presented in trend order. Bars represent mean ± SD; individual data points are shown. Data were analyzed using a two-tailed Student’s t-test with Welch’s correction. ns, not significant; **p < 0.01; ****p < 0.0001 (B).

**Figure S2. TLR4 activity, cytokine (IL-1β), and chemokine (IP-10) secretion in response to LNPs and stress factors.** (**A**) THP-1 cells (50,000/well) were exposed to media, IFNβ, IFNγ, or TNF-α (20 ng/mL) for 1 h before addition of eGFP LNPs (1 μg/mL). Following a further 48 h incubation, IL-1β levels in supernatants were measured using a DuoSet ELISA kit. Data were obtained from n ≥ 3 independent experiments, individual data points represent technical replicates pooled across experiments. Data were analyzed by two-way ANOVA. (**B**) LNPs used in this study did not induce measurable TLR4 activation. A TLR4 reporter assay was performed in HEK-293 cells stably expressing human TLR4, MD-2, and CD14 after 24 h of stimulation with LNPs formulated with the ionizable lipids MC3, SM-102, or LP01 (1 μg/mL). LPS (1 ng/mL) served as a positive control. Reporter activity was determined by substrate detection using an EnVision plate reader. Bars represent mean ± SD; n = 3 independent experiments, individual data points represent technical replicates pooled across experiments. Data were analyzed by one-way ANOVA. (**C**) Impact of innate immune priming and cytotoxic stress on LNP-induced protein expression and IP-10 release in THP-1 cells. THP-1 cells (50,000/well) were treated with IFNγ (20 ng/mL) or media for 1 h, followed by treatment with digitonin (6.25 μg/mL, Digi), thapsigargin (0.25 μM, TG), PMA (250 ng/mL), or DMSO. Supernatants were collected after 48 h and assayed for IP-10 by ELISA as described. Bars represent mean ± SD; n = 2 independent experiments. Data were analyzed using Student’s t-test. **p < 0.01, ***p < 0.001, ****p<0.0001

**Figure S3. Design and predictive modeling of LNP formulations for immune response modulation.** (**A**) Raman spectroscopy analysis showed no evidence of PEG aggregation on the surface of LNPs formulated with variable PEG levels, based on the 871-941 cm⁻¹ band. Data are presented as mean ± SD, n=3. (**B**) Ternary plots show the LNP compositional landscape, with varying molar fractions of ionizable lipid (IL), phospholipid (PHL), and cholesterol (CHL), illustrating the experimental design space. (**C**) Model validation for cytokine response prediction, with scatter plots of observed versus predicted IP-10, IL-1β, and MCP-1 levels across LNP formulations. Solid lines denote the best fit with 95% confidence intervals; RMSE and R² are indicated for each regression. (**D-G**) PEG-related immunogenic effects were observed in C14 PEG-containing LNPs but not C18 PEG or PEG lipid added directly to media. THP-1 Dual cells (50,000/well) were stimulated with IFNγ (D, F) or LPS (E, G) for 1 h or 4 h, respectively, before treatment with LNPs incorporating the standard amount of either PEG-DMG (C14, DMG) or PEG-DSG (C18, DSG) (1x, 1.5% of total lipids) or half that amount (0.5x) at a final concentration of 1 μg/mL for 48 h. Alternatively, PEG lipids (PEG-DMPE, PEG-DMG, and PEG-DSG) were dissolved directly in tissue culture media and applied to pre-stimulated THP-1 cells at a final concentration of 25 μg/mL (F, G). In each case, a condition without LNPs or PEG lipids was used as a negative control (-). IP-10 (D, F) or IL-1β (E, G) in supernatants was quantified by ELISA. Data are presented as mean ± SD and were compiled from n = 2 independent experiments. Data were analyzed by one-way ANOVA. *p<0.05, ****p<0.0001

**Supplementary Table S1.** mRNA-LNP composition and characterization data. Average size (Z-agv) and PDI were measured using Malvern ZetaSizer. PDI=polydispersity index, EE=Encapsulation Efficiency.

| LNP code | Composition [Mol %] | | | | Z-Avg [nm] | PDI | EE  [%] | N:P ratio |
| --- | --- | --- | --- | --- | --- | --- | --- | --- |
|  | Ionizible Lipid | DSPC | Cholesterol | PEG |  |  |  |  |
| MC3 | 50.0 | 10.0 | 38.5 | 1.5 | 59.3 | 0.040 | 96.0 | 6 |
| LP01 | 50.0 | 10.0 | 38.5 | 1.5 | 73.0 | 0.002 | 95.0 | 6 |
| SM-102 | 50.0 | 10.0 | 38.5 | 1.5 | 71.2 | 0.007 | 91.0 | 6 |
| SM-102 1X | 50.0 | 10.0 | 38.5 | 1.5 | 69.6 | 0.019 | 97.2 | 6 |
| SM-102 0.25X | 50.6 | 10.1 | 38.9 | 0.4 | 148.4 | 0.011 | 95.0 | 6 |
| SM-102 0.5X | 50.4 | 10.1 | 38.8 | 0.8 | 93.6 | 0.025 | 96.0 | 6 |
| SM-102 2X | 49.3 | 9.9 | 37.9 | 3.0 | 58.3 | 0.087 | 92.0 | 6 |
| SM-102 4X | 47.9 | 9.6 | 36.8 | 5.7 | 45.7 | 0.148 | 96.3 | 6 |
| SM-102 DoE #1 | 44.5 | 5.0 | 50.0 | 0.5 | 123.3 | 0.041 | 93.6 | 6 |
| SM-102 DoE #2 | 42.0 | 50.0 | 5.0 | 3.0 | 79.5 | 0.254 | 74.4 | 6 |
| SM-102 DoE #3 | 20.0 | 27.0 | 50 | 3.0 | 57.3 | 0.113 | 79.7 | 6 |
| SM-102 DoE #4 | 46.0 | 17.5 | 36.0 | 0.5 | 93.4 | 0.009 | 93.0 | 6 |
| SM-102 DoE #5 | 20.0 | 31.6 | 48.0 | 0.5 | 74.0 | 0.051 | 93.1 | 6 |
| SM-102 DoE #6 | 60.0 | 26.6 | 10.4 | 3.0 | 50.9 | 0.076 | 77.5 | 6 |
| SM-102 DoE #7 | 48.4 | 36.7 | 13.2 | 1.8 | 56.4 | 0.034 | 86.8 | 6 |
| SM-102 DoE #8 | 60.0 | 5.0 | 32.8 | 2.3 | 65.1 | 0.021 | 88.1 | 6 |
| SM-102 DoE #9 | 30.8 | 17.6 | 50.0 | 1.7 | 62.8 | 0.088 | 93.1 | 6 |
| SM-102 DoE #10 | 60.0 | 16.8 | 22.7 | 0.5 | 99.8 | 0.023 | 84.8 | 6 |
| SM-102 DoE #11 | 42.0 | 5.0 | 50.0 | 3.0 | 55.6 | 0.158 | 95.6 | 6 |
| SM-102 DoE #12 | 34.7 | 50.0 | 14.8 | 0.5 | 128.3 | 0.208 | 23.2 | 6 |
| SM-102 DoE #13 | 30.3 | 37.6 | 29.1 | 3.0 | 77.1 | 0.195 | 73.1 | 6 |
| SM-102 DoE #14 | 60.0 | 34.5 | 5.0 | 0.5 | 97.6 | 0.002 | 78.0 | 6 |
| SM-102 DoE #15 | 20.0 | 50.0 | 28.0 | 2.0 | 91.3 | 0.097 | 64.5 | 6 |
| SM-102/DoE | 40.8 | 8.7 | 50.0 | 0.5 | 129.3 | 0.017 | 85.3 | 6 |
| SM-102 0.5X DMG | 50.4 | 10.1 | 38.8 | 0.8 | 105.8 | 0.022 | 94.9 | 6 |
| SM-102 1X DMG | 50.0 | 10.0 | 38.5 | 1.5 | 81.5 | 0.074 | 96.3 | 6 |
| SM-102 0.5X DSG | 50.4 | 10.1 | 38.8 | 0.8 | 122.5 | 0.002 | 89.1 | 6 |
| SM-102 1X DSG | 50.0 | 10.0 | 38.5 | 1.5 | 90.8 | 0.091 | 97.3 | 6 |
